# Adherence in children with growth hormone deficiency treated with r-hGH and the easypod™ device

**DOI:** 10.1007/s40618-016-0510-0

**Published:** 2016-07-12

**Authors:** S. Loche, M. Salerno, P. Garofalo, G. M. Cardinale, MR Licenziati, G. Citro, M. Caruso Nicoletti, M. Cappa, S. Longobardi, M. Maghnie, R. Perrone

**Affiliations:** 1 SSD of Pediatric Endocrinology, Pediatric Hospital Microcitemico Antonio Cao AOB Cagliari, Via Edward Jenner, 09121 Cagliari, Italy; 2 Department of Translational Medical Sciences, University of Naples Federico II, Naples, Italy; 3 Endocrinology Unit, AOOR Villa Sofia-Cervello, Palermo, Italy; 4 Paediatric Division, Hospital F Ferrari, Casarano, Italy; 5 Department of Pediatrics, AORN Santobono-Pausilipon, Naples, Italy; 6 Endocrinology Unit, Mother Theresa of Calcutta Territorial Specialist Centre, Potenza, Italy; 7 Paediatric Endocrinology Service, University Hospital of Catania, Catania, Italy; 8 University-Hospital Department, Bambino Gesù Children’s Hospital, IRCCS, Rome, Italy; 9 Merck KGaA, Darmstadt, Germany; 10 Department of Paediatrics, IRCCS Giannina Gaslini Institute, University of Genova, Genoa, Italy; 11 Merck Serono S.p.A, Rome, Italy

**Keywords:** Growth disorders (GD), IGF-1, Easypod™, Adherence

## Abstract

**Purpose:**

Poor adherence to recombinant human growth hormone (r-hGH) therapy is associated with reduced growth velocity in children with growth hormone deficiency (GHD). This twelve-month observational study was to assess adherence in r-hGH patients treated with the easypod^™^, an electronic, fully automated injection device designed to track the time, date and dose administered.

**Methods:**

Ninety-seven prepubertal patients receiving r-hGH therapy were included in the study from ten Italian clinical sites and 88 completed the study. To avoid possible confounding effects, only GHD patients (79/88; 89.7 % of the overall study population) were considered in the final analysis. The primary endpoint—adherence to treatment—was calculated as the proportion of injections correctly administered during the observational period out of the expected total number of injections. The relevant information, tracked by the easypod^™^, was collected at months 6 (V1) and 12 (V2) after baseline (V0). At study termination, adherence data were partially available from 16 patients and fully available from 53 patients. As secondary endpoints, serum IGF-1 levels, fasting serum glucose and insulin levels and key anthropometric characteristics (height, waist circumference and BMI) were also determined.

**Results:**

The easypod^™^ data showed that 56.7 % of the patients were considered to be fully (≥92 %) adherent to their treatment throughout the period V0–V2. Treatment improved stature, significantly increased IGF-1 and produced a non-significant increase in blood glucose and insulin levels.

**Conclusions:**

The injection-recording system and other characteristics of easypod^™^ could enhance the ability of physicians to monitor adherence to r-hGH treatment.

## Introduction

Growth hormone (GH) has been used as an elective treatment for severely GH-deficient children and adolescents since the 1960s [1]. Due to the limited availability of human pituitary-derived hormone, GH use for other conditions related to short stature could not be seriously considered until the mid-1980s, when recombinant human GH (r-hGH) became available, thus opening up access to GH treatment to children and adolescents with causes of short stature other than GH deficiency (GHD) [2]. The indications for r-hGH in Italy are limited to the following conditions [3]: GHD, growth failure in girls with gonadal dysgenesis (Turner Syndrome), growth failure in prepubertal children due to chronic renal failure (CRF) and failure of growth in short children born small for gestational age (SGA) [4]. The marketing authorisation (MA) of some marketed products has been extended to additional indications (e.g. Prader–Willi syndrome and short stature associated with altered function of the SHOX gene) [4].

The need for frequent injections over a long period of time has stimulated research into easier methods of administration, to improve patients’ adherence to their therapy. Non-adherence, as well as low adherence, is unavoidably associated with both individual and social treatment failures, such as less favourable clinical outcomes, lower quality of life and higher healthcare costs [5].

Several devices for r-hGH administration have been developed over time. So far, five broad categories of GH injection device are available, including syringes with needle, injection pens, self-injection pens, needle-free devices and electronic devices. As reported in a recent survey by patients, parents, physicians and nurses who had experience with administration of r-hGH, an optimal r-hGH device should fulfil the following characteristics: reliability; ease of use; lack of pain during injection; safety in use and storage and minimum number of steps before injection preparation. In addition, a good tracking system, allowing effective and objective monitoring of treatment adherence, was considered extremely important by the physicians [6–10].

## Materials and methods

This was an observational, prospective study with the primary objective of monitoring adherence to r-hGH treatment for 1 year in prepubertal patients with growth disorders. The patients enrolled all received r-hGH therapy with the easypod™ Clinical Kit, a system comprising an electronic, automated injection device (easypod^™^) with a docking station for recording r-hGH administration data to enable objective monitoring of actual drug usage. The secondary objectives were to monitor the effect of r-hGH treatment on serum IGF-1 concentrations, fasting serum glucose and insulin and on anthropometric characteristics (height, waist circumference and BMI).

The first patient was enrolled on the 19th of March 2010; the last study visit was performed on the 28th of January 2013. The study was approved by the ad hoc local ethics committees, and informed consent was obtained by patient’s parents or legal guardians. Eligibility for the study was based on the following inclusion criteria:

1. Prepubertal patients with short stature (under 14 years of age) with growth disorders receiving r-hGH therapy (according to the local SmPC), who were either naïve or unsatisfied with their current device and were candidates to continue r-hGH therapy with easypod^™^ according to clinical practice;
2. Receiving r-hGH prescribed according to the local SmPC;
3. Written informed consent obtained from the parent(s)/legal guardian(s) at the beginning of the study.

Exclusion criteria included acquired GHD due to CNS tumour, infection, pituitary infiltration, history of cranial or spinal irradiation or cranial surgery; previous treatment with corticosteroids, except for topical or inhaled administration for atopic disease and/or for hormonal substitution at a stable dosage for at least 3 months and concomitant significant diseases.

Approximately 100 patients were estimated as a suitable sample size for the assessment of their adherence to treatment. Due to a slow recruitment rate, enrolment was stopped at the attainment of 97 participants. After one screening failure, a total of 96 patients were actually recruited. Eight patients did not complete the observational period for various reasons, so 88 patients completed the study.

Due to the small number of patients with conditions other than GHD, and in order to avoid possible confounding effects, only the 79 patients with GHD were included in the analysis data set. Patients could elect to discontinue their participation at any time. The disposition of the patients is summarised in Table 1.

**Table 1:** Patient disposition

Status	Frequency	Percentage
Screened	97	100
Screening failure	1	1
Enrolled	96	99
Early termination	8	8
Completed	88	92
Analysis data set (only GHD patients	79	82

**Fig. 1:**
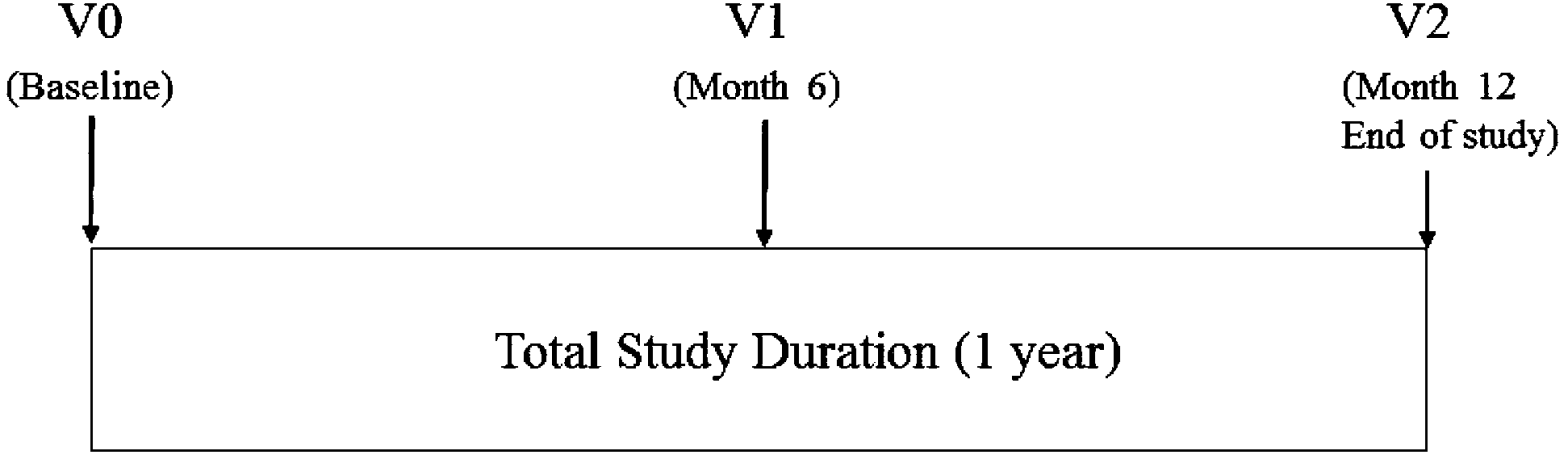
Study design and plan

The study design is summarised in Fig. 1:

The study duration for each recruited patient was 1 year, unless they discontinued prematurely.

Each patient was enrolled in the study at baseline visit, after the assessment of eligibility criteria. Easypod^™^ devices were supplied by Merck Serono SpA, sponsor of the study. A support service, provided by the sponsor, was guaranteed to the enrolled patients in order to train them in correct device usage and replacement procedures, should malfunction of the device occur during the study.

Pursuant to the observational nature of the study, the r-hGH treatment was administered according to routine clinical practice, independent of the patient’s participation in the study. The outcome measurements for both the primary and the secondary endpoints were assessed at visits V0, V1 and V2, respectively. The adherence to the treatment for each patient was estimated as the proportion of injections correctly administered during the observational period out of the expected total number of injections. The target rate for full adherence was defined as ≥92 % at the start of the study.

Adherence was calculated only for patients reporting at least 150 injections every 6 months (at least 300 injections throughout the overall 12-month observational period). The adherence rate was calculated as follows:

$$ {\text{Treatment adherence rate (}}\% ) { } = \frac{\text{Number of days injections received during period}}{\text{Number of days injections planned during period}}\, \times 100 $$

The statistical analysis was performed with SAS^®^ software, version 9.2 (SAS Institute, Cary, NC, USA). As this was an observational study, no distinction between intention to treat and per protocol data sets was made, no subsets were identified and only descriptive statistical analysis was performed. IGF-1 concentrations were measured by local laboratories using standard assays. IGF-1 SDS was calculated using the normative data for the method [11].

## Results

Within an overall study population composed of 88 enrolled subjects, 79 patients, all with GHD, were included in the final analysis. Of these, 52 (66 %) were male and 27 (34 %) were female. Median age at enrolment was 10 years (interquartile range 9–12).

### Adherence to treatment

Adherence data were available from 53/79 (67.09 %) participants for the whole 12-month study period and from 16/79 (20.25 %) patients for a 6-month follow-up (either from V0–V1 or from V1–V2). Only 16 patients reported ≥150 injections over 6 months between V0–V1 and V1–V2. Overall, 30/53 patients reported a total number of injections ≥300 across the whole observation period. Easypod^™^ data showed that 17/30 (56.67 %) patients administering at least 300 injections across the 1-year follow-up period completed the study with the preset target adherence rate 92 %. With respect to the length of the follow-up period, administration data collected through the easypod^™^ were available from 28/53 (52.83 %) adherent patients. Relevant results are summarised in Tables 2 and 3.

**Table 2:** Adherence by number of injections

	Adherence by number of injections (V0–V2)			
	Adherence rate	Number of injections		
		<300	≥300	Total
Frequency (%)	<92 %	12 (22.64 %)	13 (24.53 %)	25 (47.17 %)
Frequency (%)	≥92 %	11 (20.7 %)	17 (32.1 %)	28 (52.8 %)
Total (%)		23 (43.4 %)	30 (56.6 %)	53 (100 %)

**Table 3:** Adherence by follow-up period

	Adherence	Number of subjects (%)
Between V0 and V1	Not adherent	29 (45.3)
	Adherent	35 (54.7)
Between V1 and V2	Not adherent	24 (41.4)
	Adherent	34 (58.6)
Between V0 and V2 (whole study period)	Not adherent	25 (47.2)
	Adherent	28 (52.8)

Changes from baseline of height SDS were evaluated, showing a significant increase in height across the 12 months of follow-up (Table 4).

**Table 4:** Changes from baseline in height SDS

Changes from baseline	*n*	Mean	Standard deviation	Median	Interquartile range	*p* value
Height SDS V0	77	−2.2	0.8	−2.2	−2.67 to −1.79	
Height SDS V2	76	−1.7	0.7	−1.7	−2.33 to −1.27	
Height SDS V2–V0	75	0.5	0.3	0.5	0.25–0.64	<0.0001

No correlation was found, using a linear regression model, between the change in height SDS among fully adherent patients (300 injections in the whole period) and the adherence rate (coefficient *β* = 0.01241, *p* = 0.123).

Serum glucose and insulin concentrations increased slightly, but not significantly, from baseline to V1 and V2, while IGF-1 significantly increased, as expected (Table 5).

**Table 5:** Changes from baseline in IGF-1 blood glucose and insulin levels

Changes from baseline and between visits	*n*	Mean	Standard deviation	Median	Interquartile range	*p* value
IGF-1 V0 (ng/mL)	67	204	129	183	116–256.3	
IGF-1 V1 (ng/mL)	52	278	136	275	185.5–356.75	
IGF-1 V1–V0	50	89	131	77	19–154	<0.0001
IGF-1 V2 (ng/mL)	63	290	143	292	162–374	
IGF-1 V2–V0	54	97	148	88	42 – 158	<0.0001
Blood glucose V0 (mg/dL)	40	82	9	81	77–87	
Blood glucose V1 (mg/dL)	56	86	9	87	81–92	
Blood glucose V1–V0	39	2	9	1	−4 to 8	0.2262
Blood glucose V2 (mg/dL)	63	89	42	87	80–91	
Blood glucose V2–V0	38	3	11	2	−5 to 9	0.2043
Insulin V0 (μU/mol)	37	6	5	5	3–8.25	
Insulin V1 (μU/mol)	45	7	4	7	3.7–9.5	
Insulin V1–V0	29	1	4	1	−0.9 to 4	0.1459
Insulin V2 (μU/mol)	51	8	6	7	4.99–10	
Insulin V2–V0	28	1	4	1	−0.76 to 3.8	0.1628

Assessment of anthropometric characteristics revealed a statistically significant increase in height both at visit V1 and V2 (Table 6).

**Table 6:** Changes from baseline in height parameters

Changes from baseline and between visits	*n*	Mean	Standard deviation	Median	Interquartile range	*p* value
Height V0 (cm)	79	126	16	129	118.7–136.2	
Height V1 (cm)	79	130	16	133	122.3–141	
Height V1–V0	79	4	2	4	3.3–5.5	<0.0001
Height V2 (cm)	79	134	15	137	126.7–144.6	
Height V2–V0	79	8	2	8	6.7–9.5	<0.0001

**Fig. 2:**
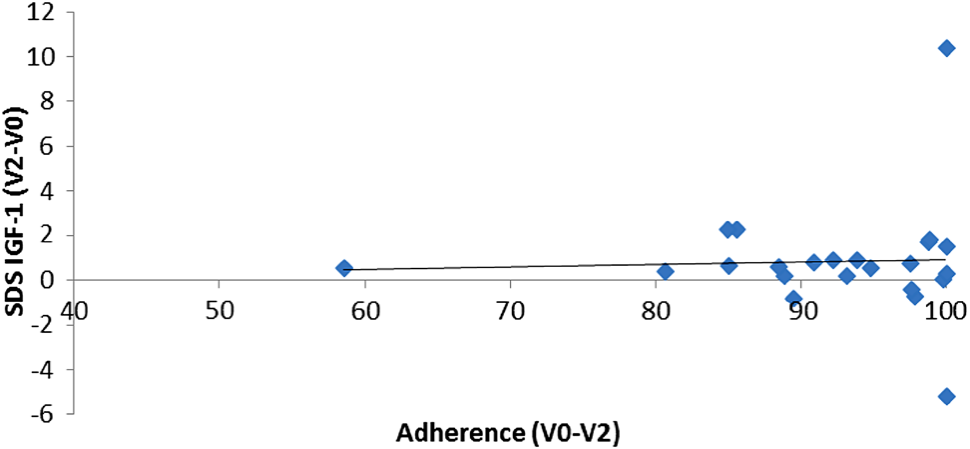
Linear regression IGF-1 SDS/adherence

A linear regression model showed no relationship between the changes observed in IGF-1 standard deviation score (IGF-1 SDS) and the adherence rate of patients with at least 300 injections in the whole period (coefficient *β* = 0.01122, *p* = 0.8517). Detailed results are summarised in Fig. 2.

## Discussion

In this study, we report the adherence rate measured by easypod in the 53 of 79 prepubertal patients with GHD (52 [66 %] boys, 27 [34 %] girls) for whom data were available, who had completed >300 injections over 12 months. Demographic characteristics of the patients included were consistent with the usual profile of easypod^™^ Clinical Kit users. The majority of patients showed good adherence to treatment, better than that reported in previous studies [12, 13]. However, it must be stated that our study was prospective, and we used an objective method to measure adherence. In addition, it must be pointed out that the patients (and their parents) were aware that adherence to treatment was being monitored and this fact may have influenced the outcome. We found no correlation between change in height SDS and the adherence rate. This may be due to the fact that the great majority of patients had a high adherence rate, and the number of patients was too small to find a correlation with a parameter which varies very little.

As expected, we found a slight but non-significant increase in serum insulin and glucose concentrations. A number of studies have shown that such an increase has no clinical significance [14, 15]. IGF-1 significantly increased but always remained within the normal range of concentrations.

The data collected by easypod^™^ Clinical Kit showed that 56.67 % of patients were fully adherent (adherence 92 %) to treatment for the whole period of observation (V0–V2). Our results did not show a relationship between the changes observed in IGF-1 SDS and the adherence rate of patients with at least 300 injections in the whole period. Notably, IGF-1 concentrations significantly increased in the whole studied population both at V1 and at V2. Consequently, IGF-1 concentrations were increased to therapeutic levels in the overall population. Further studies are needed, either to specifically assess the minimum number of injections necessary to achieve a therapeutic effect or to compare IGF-1 SDS levels between adherent and non-adherent patients.

Adherence to treatment has been demonstrated to be critical for the achievement of both medical and economic expected outcomes of GH therapy [16–18]. However, Fisher et al. [18] have shown that non-adherence to GH therapy in paediatric patients is affected by several factors, among which the adoption of a needle-free injection device in place of a multi-dose injection pen may not play a crucial role, according to Verrips et al. [19]. Nevertheless, Cutfield et al. [20] have pointed out how subjective (parent-reported) and objective (empty vial count) adherence rates may differ from one another, thus demonstrating unequivocally the usefulness of a device, like the easypod™, that is able to collect objective data.

## Conclusions

The results of the present study suggest that easypod^™^ may represent a helpful option that could assist physicians in effective monitoring of adherence to r-hGH treatment. The treatment resulted in significant changes in height SDS and IGF-1 concentrations. Further studies are needed to compare growth and IGF-1 levels between adherent vs non-adherent patients. Nevertheless, our study demonstrates that, even when therapeutic adherence is not strictly observed, but IGF-1 levels are maintained at therapeutic levels, with modern, easy to use, recombinant hormone formulations, r-hGH supplementation is safe and efficacious.
